# Lactate drives cellular DNA repair capacity: Role of lactate and related short-chain fatty acids in cervical cancer chemoresistance and viral infection

**DOI:** 10.3389/fcell.2022.1012254

**Published:** 2022-10-19

**Authors:** Wojciech M. Ciszewski, Katarzyna Sobierajska, Anna Stasiak, Waldemar Wagner

**Affiliations:** ^1^ Department of Molecular Cell Mechanisms, Medical University of Lodz, Lodz, Poland; ^2^ Department of Hormone Biochemistry, Medical University of Lodz, Lodz, Poland; ^3^ Laboratory of Cellular Immunology, Institute of Medical Biology, Polish Academy of Sciences, Lodz, Poland

**Keywords:** lactate, SCFA, DNA repair, NHEJ, ABCB1, cancer, virus

## Abstract

The characteristic feature of a cancer microenvironment is the presence of a highly elevated concentration of L-lactate in the tumor niche. The lactate-rich environment is also maintained by commensal mucosal microbiota, which has immense potential for affecting cancer cells through its receptoric and epigenetic modes of action. Some of these lactate activities might be associated with the failure of anticancer therapy as a consequence of the drug resistance acquired by cancer cells. Upregulation of cellular DNA repair capacity and enhanced drug efflux are the most important cellular mechanisms that account for ineffective radiotherapy and drug-based therapies. Here, we present the recent scientific knowledge on the role of the HCA1 receptor for lactate and lactate intrinsic activity as an HDAC inhibitor in the development of an anticancer therapy-resistant tumor phenotype, with special focus on cervical cancer cells. In addition, a recent study highlighted the viable role of interactions between mammalian cells and microorganisms in the female reproductive tract and demonstrated an interesting mechanism regulating the efficacy of retroviral transduction through lactate-driven modulation of DNA-PKcs cellular localization. To date, very few studies have focused on the mechanisms of lactate-driven enhancement of DNA repair and upregulation of particular multidrug-resistance proteins in cancer cells with respect to their intracellular regulatory mechanisms triggered by lactate. This review presents the main achievements in the field of lactate impact on cell biology that may promote undesirable alterations in cancer physiology and mitigate retroviral infections.

## 1 Introduction

The cancer tumor and the cancer microenvironment have been recently presented as two-compartmental diseases that mutually drive processes to sustain cancer energy demand, shape tumor vascularization, and attenuate host immune system response. The characteristic feature of a settled cancer niche is the presence of high concentrations of L-lactate in the tumor microenvironment area (even 40 mM), which is regarded as a protumorigenic stimulus (Hirschhaeuser et al., 2011). Accumulated evidence demonstrates that aberrant glucose metabolism of cancer cells and its product lactate may prime changes in cellular DNA repair mechanisms often correlated with antineoplastic treatment failures rather than success (Bhatt et al., 2015; Balboni et al., 2021; Govoni et al., 2021; Naik and Decock, 2022). Thus, the emerging role of this molecule in the development of cancer cells, chemo- and radio-resistance (Roland et al., 2014; Wagner et al., 2015; Wagner et al., 2017a; Wagner et al., 2017b; Xie et al., 2017), and rearrangement of the host immune response has been raised and has drawn the attention of oncologists (Yabu et al., 2011; Wagner et al., 2016; Feng et al., 2017). Parallelly, the human microbiota has been recognized as an essential source of small modulatory molecules, including lactate and relative short-chain fatty acids (SCFA). However, their substantial supportive physiological role is still being unveiled (Lee et al., 2018; Martin-Gallausiaux et al., 2021). Indeed, non-tumor-related high-lactate environment is normally present in the gut, vagina, and oral cavity, where commensal lactic acid bacteria extensively metabolize carbohydrates to lactate (Englander et al., 1959; Domingue et al., 1991; Lee et al., 2018). A high concentration of lactate is the exclusive feature of the lower female genital tract (FGT). Vaginal secretions may contain up to 50 mM of lactate (Domingue et al., 1991). Furthermore, lactic acid bacteria of the oral cavity are responsible for short-term elevated levels of lactic acid in the teeth plaque after sugar ingestion, exceeding, over 4-fold, the lactate concentration observed in FGT (Englander et al., 1959). On the contrary, in fecal samples from healthy donors, lactate produced by gut microbiota is immediately further metabolized within the colon; however, inflammatory conditions may significantly elevate lactate concentrations over 30-fold (Belenguer et al., 2007). Here, we review the recent advances in knowledge about lactate and its effects on DNA repair mechanisms. We focus on the lactate-driven changes in the expression and translocation of DNA repair enzymes, involvement of lactate signaling through HCA1 receptor, and the role of monocarboxylate- and ABC transporters in resistance to chemotherapeutics. Finally, we discuss how these molecular alterations can lead to the mitigation of retroviral infections by cervical epithelium and thus restrict the retrovirus spread.

## 2 Lactate metabolic pathways in human and associated microbiota

**FIGURE 1:**
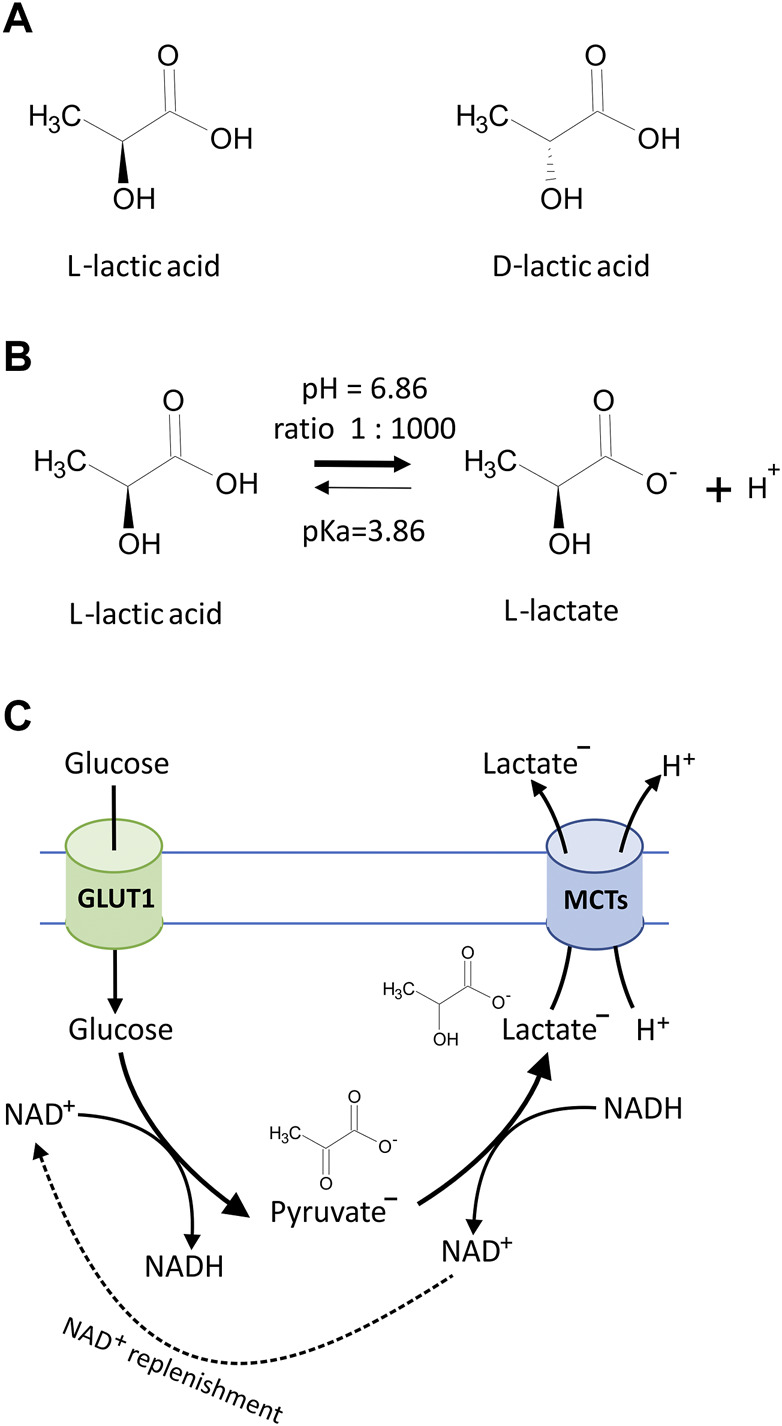
**(A)** Chemical structures of lactic acid isomers, L-lactic acid and D-lactic acid. **(B)** Lactic acid is a weak organic acid with pKa = 3.86, and in biological systems, lactic acid undergoes ionization to produce carboxylate anion CH3CH(OH)COO^−^ (lactate) and H^+^. Under cellular physiological pH (7.0–7.4), the lactate to lactic acid ratio exceeds 1,000:1 according to the Henderson–Hasselbalch equation, which describes the mathematical relationship between the pH value, the log of the acid dissociation constant (pKa), the molarity of the acid, and the molarity of the base [pH = pKa + log_10_ (conjugate base/weak acid)]. **(C)** The simplified diagram of lactate generation and excretion in eukaryotic cells. Glucose transport across the cell membrane is facilitated by glucose transporters (GLUT1). In the cytosolic compartment of the cell, glucose is converted to pyruvate *via* a series of intermediate metabolites to yield a net of 2 molecules of adenosine triphosphate (ATP) and reduced nicotinamide adenine dinucleotide (NADH). Cells carrying anaerobic respiration (hypoxic conditions and tumor cells) subsequently reduce pyruvate to lactate and thus regenerate the pool of NAD^+^. Excretion of resulting lactate along with protons (H^+^) is facilitated by monocarboxylate transporters (MCTs) according to substrate and H^+^ concentration gradients.

**FIGURE 2:**
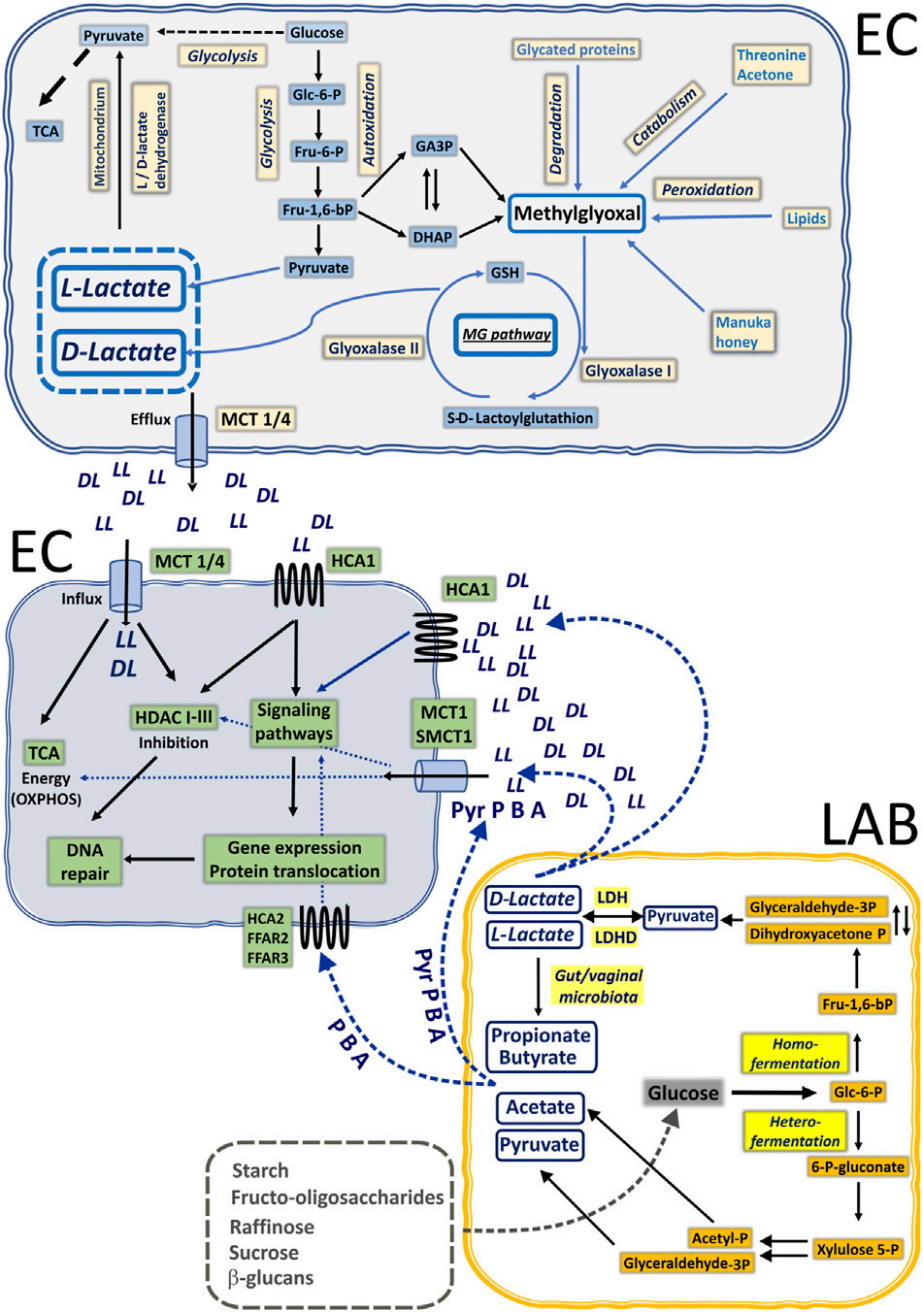
Illustration of lactate and SCFA biogenesis in the common mucosal niche of epithelial cells (EP) and lactic acid bacteria (LAB) in humans. Under anaerobic conditions, glucose is preferentially catabolized to L-lactate (LL) *via* glycolysis. Cancer cells also utilize glucose to make L-lactate in the presence of sufficient oxygen *via* aerobic glycolysis. Boosted glycolysis pathway generates highly reactive intermediates detoxified through methylglyoxal pathway to D-lactate. Generated L- and D-lactate (DL) can be used to replenish the pyruvate pool for TCA cycling or be exported *via* monocarboxylate transporters (MCT1-4) to extracellular space. Healthy commensal microbiota of gastrointestinal and urogenital tracts are able to produce lactate and short-chain fatty acids through homo- and heterofermentation of glucose. Locally produced lactate by EP cells and lactate together with butyrate (B), acetate (A), and propionate (P) by LAB can trigger neighboring EP cells through specific GPCRs: hydroxycarboxylic acid receptors (HCA1/2) and free fatty acid receptors (FFAR2/3). Alternatively, monocarboxylic acids can be actively taken up by EP *via* MCTs or SMCT1 and exert their intracellular inhibitory action on HDACs or to fuel the TCA cycle (lactate and pyruvate, Pyr).

In normal cells, glucose is actively taken up by cellular glucose transporters and follows consecutive enzymatic steps to yield pyruvate and two molecules of ATP. This preliminary step, known as glycolysis, does not require oxygen. In the anaerobic (hypoxic) condition, the pyruvate stays in the cytoplasm and glucose is preferentially catabolized to lactate as the source of L-lactate (Figures 1, 2). Under aerobic conditions, the product of glycolysis (pyruvate) is further transported to the mitochondria to undergo oxidative phosphorylation (OXPHOS). After initial oxidization of pyruvate by the pyruvate dehydrogenase (PDH) complex, the resulting acetyl coenzyme A (CoA) follows a series of consecutive chemical reactions known as tricarboxylic acid (TCA) cycles. Finally, the combined chain of glycolysis, TCA, and oxidative phosphorylation pathways yields a net of 36 molecules of ATP from the oxidation of one molecule of glucose. In the early 20th century, Otto Warburg discovered high glucose uptake and preferential lactate production in cancer cells to support their high ATP demand. These distinctive features of the Warburg effect, namely, the high glycolysis rate and low OXPHOS, are advantageous for highly proliferating tumor cells (Liberti and Locasale, 2016). Apart from aerobic glycolysis, tumor cells could also replenish the lactate pool on demand by engaging in glutamine catabolism, known as glutaminolysis (DeBerardinis et al., 2007). Rapid cellular L-lactate build-up can be excreted by monocarboxylate transporters (MCTs), which are essential for the survival of cancer cells. MCTs, namely, MCT1 and MCT4, are the key plasma membrane transporters that maintain two-way transport of L-lactate together with H^+^ according to substrate and proton concentration gradients. Paradoxically, L-lactate shuttling or feeding is commonly observed between cancer hypoxic and normoxic sites (Goodwin et al., 2015) and between astrocytes and neurons with the highest energy needs (Dienel, 2019). In this form of metabolic symbiosis, L-lactate may be used as a primary energetic molecule, sparing glucose for highly glycolytic cells. Highly glycolytic cancer cells in the hypoxic area of the tumor produce and export lactate through a low-affinity MCT4, while tumor cells supplied with oxygenated blood abundantly express a high affinity MCT1 for lactate to maintain efficient monocarboxylate influx and feed their oxidative phosphorylation pathway (Goodwin et al., 2015). In turn, MCT1 is involved in lactate efflux in astrocytes to efficiently feed neurons with lactate as an energy substrate (Powell et al., 2020). Apart from the bioenergetic role of L-lactate, this molecule exhibits low inhibitory effects on intracellular histone deacetylases (HDAC) that result in protein modifications such as acetylation (histones, HMGB1) and lactylation (histones, MAPK, citrate synthase, eIF5a) (Chen et al., 2021; Yang et al., 2022). Furthermore, in the extracellular compartment, L-lactate acts as a hormone through its specific surface receptor HCA1 (aka GPR81), expressed on various normal and cancer cells (Ahmed, 2011; Brown and Ganapathy, 2020). All these unique features of lactate enable this molecule to reshape cellular metabolism and mimic aspects favorable for tumor development under hypoxic conditions. An elevated L-lactate level in the tumor niche, as a result of metabolic reprogramming through the rapamycin (mTOR) pathway cascade (Harachi et al., 2018) increases the expression of HIF-1α and c-Myc (Dodd et al., 2015; Liu et al., 2017). Concomitantly, these transcriptional factors are crucial players in a maintenance of cancer cell metabolism at a highly glycolytic rate *via* multiple and distinctive mechanisms (Wang et al., 2021). First of all, both HIF-1α and c-Myc participate in glucose feeding of cancer cells by upregulation of the expression of glucose transporter 1 (GLUT1) (Wang et al., 2021). In addition to glycolysis substrate transport, HIF-1α and c-Myc facilitate key steps of glycolysis by positive regulation of hexokinase 2 (HK-II), phosphofructokinase-1 (PFK-1), and pyruvate kinase M2 (PKM2) expressions (Li et al., 2020), and therefore increase the pyruvate pool for lactate formation by lactate dehydrogenase. They also upregulate pyruvate dehydrogenase kinase-1 (PDK1), trigger inhibition of pyruvate dehydrogenase *via* its phosphorylation, and thus slow-down TCA cycle entry by pyruvate, its oxidative phosphorylation, and generation of harmful reactive oxygen species (ROS) (Papandreou et al., 2006). Finally, HIF-1α and c-Myc may activate lactate dehydrogenase-5 (LDH-5), which promotes the conversion of pyruvate to L-lactate (Koukourakis et al., 2003) and concomitantly inhibit LDH-1 (that favors the conversion of pyruvate to acetyl-CoA and Krebs cycle entry), leading to cellular lactate build-up (Wang et al., 2021). An excess of cellular lactate is transported to the extracellular space along with H^+^, mostly by MCT1/MCT4 (Figure 1). In part, these processes are regulated by HIF-1α (either MCT1 or MCT4) (Ullah et al., 2006; Miranda-Gonçalves et al., 2016) as well as by c-Myc (MCT1) (Doherty et al., 2014). The extracellular distribution of L-lactate and acidification of the cancer microenvironment (by H^+^ efflux) is a widely recognized attribute of the tumor, and is essentially associated with tumorigenesis and metastasis. L-Lactic acidosis within tumor microenvironment (TME) is suppressive to the immune system, induces an anergic state in T cells, and drives cancer escape from lymphocyte infiltration (Certo et al., 2021). L-lactate, abundantly present in a tumor niche, primes the differentiation of monocytes into dendritic cells with an immunosuppressive phenotype (Gottfried et al., 2006) or into macrophages with an inflammatory protumor phenotype M2 (Colegio et al., 2014). Hypoxia is known to be involved in fibroblast reprogramming to cancer-associated fibroblasts (CAFs) *via* HIF-1α pathways. In that circumstance, cell metabolism moves to glycolysis, demonstrated by higher lactate production (Fiaschi et al., 2012; Zeng et al., 2015). The increased CAF population promotes cancer metastasis *via* facilitating angiogenesis, alteration of ECM components and induction of cancer cell invasion ability (Hielscher et al., 2013; Kai et al., 2016; Kalluri, 2016). Hypoxic and acidic conditions in the TME may also confer a more drug-resistant tumor phenotype due to enhanced expression of multidrug-resistance proteins in cancer cells. Upregulation of HIF-1α in these conditions often translates into a higher abundance of membrane ABCB1, ABCC1, and ABCG2 transporters and diminished drug efficacy as a result of its enhanced efflux (Cheng and To, 2012; Chen et al., 2014; Lv et al., 2015).

In eukaryotic cells, D-lactate is produced exclusively as the end product of methylglyoxal detoxification (Figure 2). Although methylglyoxal can be produced as a by-product of degradation of advanced glycation end products, protein, and fatty acid metabolism (Allaman et al., 2015), enhanced glycolysis is the dominant source of endogenous methylglyoxal. An elevated concentration of glucose (e.g., hyperglycemia) boosts the glycolysis pathway and accounts for increased break down of the dihydroxyacetone phosphate (DHAP) and triosephosphates glyceraldehyde-3-phosphate (GAP). Detoxification of methylglyoxal requires activity of Glyoxalase-1 (Glo-1) followed by Glyoxalase-2 (Glo-2) action. Initially, Glo-1 facilitates the formation of S-Lactoylglutathione from hemithioacetal, which was previously generated in the spontaneous reaction of methylglyoxal with reduced glutathione (GSH). In the next step, S-Lactoylglutathione is catalyzed into D-Lactate by Glo-2. Thus, when produced and accumulated, both L- and D-lactate can affect specific intracellular targets (HDACs) or be redistributed *via* MCT efflux, or employed in OXPHOS after conversion to pyruvate by L-lactate dehydrogenase (LDH) and D-lactate dehydrogenase (LDHD) (Monroe et al., 2019), accordingly.

Human commensal microbiota, especially Lactobacilli, colonize the gut, oral cavity, and female reproductive tract as soon as the infant is breast-fed. In particular, the vaginal commensal bacteria have developed *via* constant transmission of species from the intestine to the vagina. Thus, vertical transmission of microorganisms after oral administration viably influences both gastrointestinal and vaginal microbiota composition and immunity (Amabebe and Anumba, 2020). In other words, microbes and their small, biologically active mediators released in the vagina and the gut are explicitly in reciprocal action. Plant high-chain carbohydrates and poly- and oligosaccharides are utilized by lactic acid bacteria (LAB) by two main routes of substrate phosphorylation: homo- and heterofermentation (Petrova and Petrov, 2020). Homofermentative LABs (Lactococcus, *Streptococcus*, Leuconostoc, Weissella, and some *Lactobacillus* species) metabolize carbohydrates to pyruvate *via* the Embden–Meyerhoff–Parnas pathway. The presence of L-LDH and D-LDH, which are stereospecific nicotinamide adenine dinucleotide (NAD)-dependent enzymes, provides pyruvate conversion either to the L or D isomer. Under specific environmental conditions, for example, carbon shortage, the homolactic metabolism could be reoriented to the heterofermentation process, which is a mixed-acid metabolism where pyruvate, acetate, and ethanol are also produced. Some heterofermentative LABs like Leuconostoc and particular *Lactobacillus* species could employ the phosphoketolase pathway (PKP) for sugar fermentation. In this fermentation pathway of pentoses, intermediate molecules of pyruvate and acetyl-P are subsequently transformed to acetate and ethanol. In addition to LABs, anaerobic intestinal bacterial strains possessing butyryl-CoA:acetate-CoA transferase are able to further utilize acetate to butyrate, or lactate to propionate *via* succinate/acrylate/propanediol pathways (Duncan et al., 2002; Reichardt et al., 2014; Amabebe and Anumba, 2020). Thus, symbiotic human microbiota through mutual interactions can produce a large number of small, biologically active compounds, namely, lactate and SCFA: butyrate, acetate, and propionate (Parada Venegas et al., 2019; Amabebe and Anumba, 2020) that can exert profound local and distant effects on host systems, either directly or *via* specific surface receptors.

## 3 Cellular mechanisms for lactate sensing and lactate intracellular delivery

Eukaryotic cells sense the external environment mostly *via* specific membrane metabotropic receptors. These sensors principally include G-protein-coupled receptors (GPCR) and receptor tyrosine kinases (RTKs), and a number of them (e.g., LPAR1, AT1R, IGF-1R, EGFR) have an established role in cancer development and spread (Xu and Huang, 2010; Liu et al., 2016). Recently, the special attention of scientists has been attracted by small, biologically active compounds, namely, lactate, butyrate, acetate, and propionate as they are produced locally and can be sensed by target cells in distant tissues *via* specific surface receptors: HCA1 (GPR81), HCA2 (GPR109A), FFAR2 (GPR43), and FFAR3 (GPR41), respectively (Carretta et al., 2021). Interestingly, L-lactate has been attributed to the function of a hormone acting *via* HCA1 in an autocrine or paracrine fashion. The list of lactate effectory cells bearing HCA1 includes adipose tissue cells (Liu et al., 2009), brain cells (Bozzo et al., 2013; Morland et al., 2015), tumor cells (Roland et al., 2014; Wagner et al., 2015), among others. The receptor for lactate, namely, hydroxycarboxylic acid receptor 1 (HCA1), belongs to the GPCR family that couples to Gi protein. Three natural ligands for HCA1 have been recognized so far: L-lactate, D-lactate, and 3,5-dihydroxybenzoic acid (DHBA) (Li et al., 2014; Wagner et al., 2015). After lactate or DHBA stimulation, activated HCA1 inhibits adenylyl cyclase and related cAMP pathways while initiating the PKC/MAPK pathway in a Pertussis toxin-sensitive fashion (Li et al., 2014). Particularly, the inhibition of Gβγ subunit-dependent signaling by pretreatment of the skeletal muscle cells or CHO-HCA1 cells with M119K inhibitor ceased ERK1/2 activation and subsequent IGF-1R activation in the presence of HCA1 ligands (Li et al., 2014). In addition, subsequent downstream pathways including MEK1/2-ERK1/2-p90RSK axis (Ohno et al., 2018), PI3K/Akt-CREB signaling pathway (Lee et al., 2016), and arrestin-β2-dependent signaling (Hoque et al., 2014) have been described. Previously, Raychaudhuri and colleagues proposed another Gβγ subunit-dependent signaling pathway upon lactate stimulation. A study on intratumoral plasmacytoid dendritic cells showed lactate-triggered Gβγ-dependent cytosolic free Ca^2+^ mobilization followed by calcineurin phosphatase activation, resulting in type I IFN downregulation (Raychaudhuri et al., 2019).

Recent literature contains several reports describing lactate’s impact on the activity of cellular DNA repair machinery and its consequences, mostly in cancerous cervical epithelial cells (Wagner et al., 2015). These mechanisms are mediated by intracellular inhibition of histone deacetylase (HDAC), resulting in changes in nuclear chromatin compactness as well as governed by HCA1 signaling pathways, described above in detail. Monocarboxylate transporters (MCTs) coordinate cellular lactate influx and thus render its intracellular biological activity. Four monocarboxylate transporters (MCT1-4) have been identified in eukaryotic cells (Pinheiro et al., 2009; Moschen et al., 2012; Payen et al., 2020), which play a major role in two-way transport of monocarboxylates across the plasma membrane that depends on substrate and proton gradient. MCT1 and MCT4 isoforms are abundantly expressed in cancer cells and are considered as promising molecular targets for new anticancer modalities (Payen et al., 2020). Subsequently, our studies unveiled the positive role of MCTs in lactate-driven enhancement of the DNA repair capacity of cervical cancer cells (Wagner et al., 2015). Complimentary experiments with the use of a pan-MCT inhibitor, an α-CHCA, have shown that lactate-driven stimulation of cellular DNA repair machinery and enhanced cell survival are related to its intracellular activity. Interestingly, these effects were in part related to HCA1 status, as HeLa cells expressing shRNA against HCAR1 showed a significantly lower mRNA level for MCT4, compared to control shRNA-expressing cells. Similar observations of partial control of HCA1 over MCTs have also been demonstrated in numerous studies on pancreatic, breast cancer, and HSCC (Roland et al., 2014; Jia et al., 2020; Ishihara et al., 2022). Inside cells, lactate and butyrate share similar intrinsic inhibitory activities against cellular HDACs. HDACs and their opposing counterparts HATs (histone acetyltransferases) orchestrate protein acetylation in the cytosolic and nuclear compartments of the cells. Additionally, butyrate could specifically drive auto-acylation of acetyltransferase p300, thus enabling self-activation and prime chain reaction of histone/protein acetylation (Thomas and Denu, 2021). In such a model of enhanced process of histone acetylation (hyperacetylation), more relaxed and transcriptionally permissive chromatin conformation is promoted. The opposite effects are seen in the HAT/HDAC interplay, resulting in hypoacetylation of histones, translating into transcriptionally repressive chromatin states along with higher condensation states. Such characteristics have been recently reported (Latham et al., 2012; Wagner et al., 2015). Both authors confirmed that inhibition of HDAC enzymatic activity by butyrate or lactate induced a significantly higher acetylation level of histones H3 and H4, followed by a decrease in DNA compactness (Wagner et al., 2015). While butyrate is considered a strong inhibitor of HDACs, lactate is characterized by weak to moderate inhibitory activity of HDAC I/II with IC50 = 124 mM and 32 mM for L-lactate and D-lactate, respectively (Wagner et al., 2015). This study revealed lactate D isomer as a more effective molecule than L-lactate with regard to inhibition of HDAC I/II, histone H3/H4 acetylation, chromatin relaxation, and stimulation of the Erk signaling pathway. Although the difference between histone acetylation rendered by these two lactate isomers was demonstrated, these changes translated into enhanced chemoresistance of cervical cancer cells regardless of the isomer treatment (Wagner et al., 2015). Interestingly, in human lymphoma B experimental settings, D-lactate isomer could induce *IL13* expression and IL-13 secretion to a higher extent than the established strong HDAC inhibitors, for example, SAHA (suberoylanilide hydroxamic acid/Vorinostat) and butyrate (Wagner et al., 2016). Thus, lactate, a lactic acid bacteria fermentation product, could be considered as a significant player in epigenetic regulation and function of chromatin, and attributed to the regulation of gene expression, including DNA repair proteins. Collectively, such interplay among HDAC inhibitors and HATs may promote DNA repair processes as a result of an established repair-proficient chromatin state, attracting signaling and repair proteins (Wagner et al., 2015; Wagner et al., 2021).

## 4 Lactate-driven upregulation of the proteins involved in cellular DNA repair in cervical cancer cells

**FIGURE 3:**
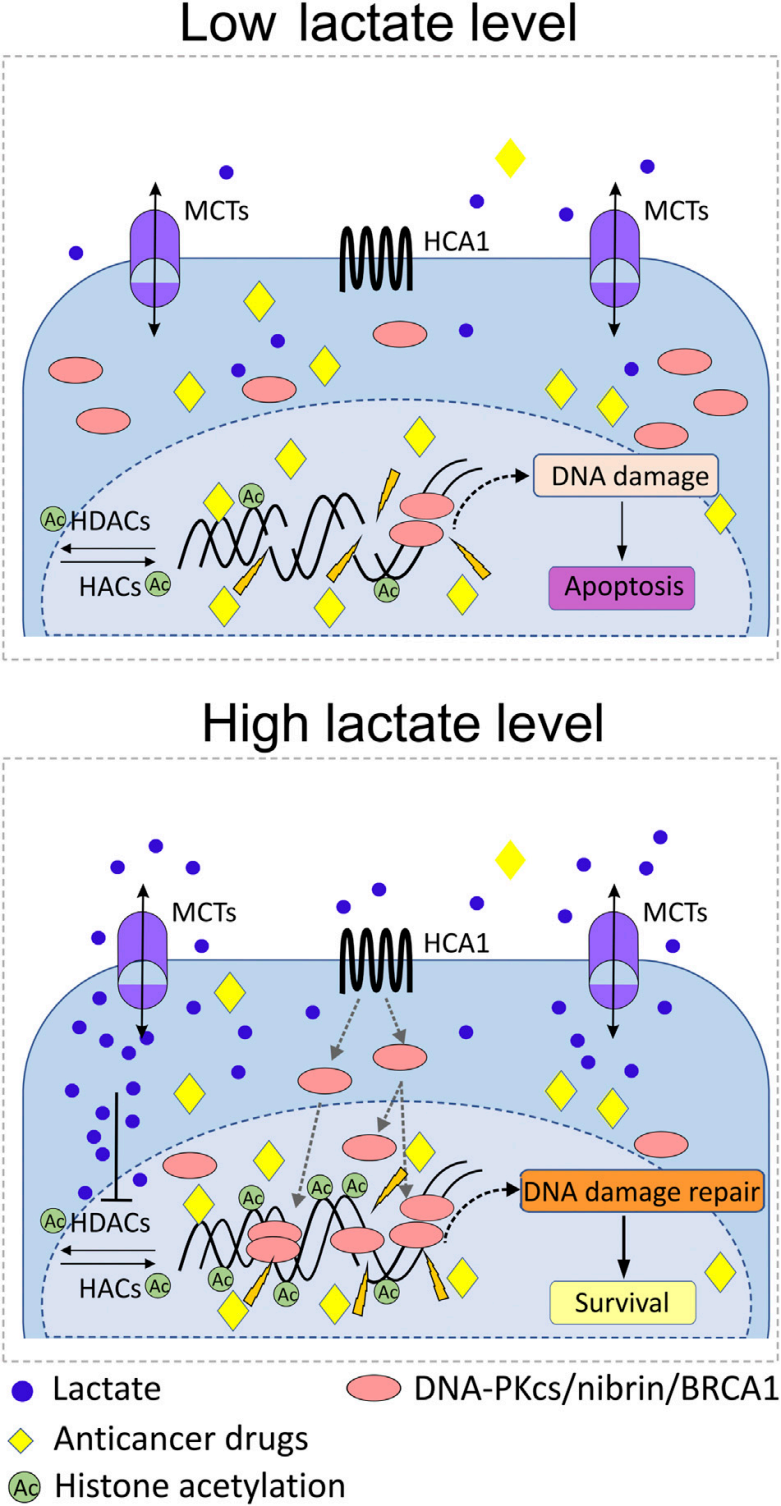
Role of lactate in the mobilization of DNA repair proteins and protection against anticancer radiotherapy or drug-based therapies. Abundance of lactate in pericellular and intracellular compartments as a result of MCT1/4-driven flux of lactate triggers upregulation of particular DNA repair proteins of NHEJ and HR systems in the nucleus, for example, DNA-PKcs, BRCA1, and nibrin. Promotion of HCA1 signaling by lactate, and acetylation of chromatin due to lactate inhibitory activity against histone deacetylases (HDACs) induce transcription of DNA repair genes and recruitment of DNA-PKcs, BRCA1, and nibrin to nuclear compartment. The lactate-rich environment accelerates processing of genomic DNA damaged by anticancer drugs/radiation and thus promotes the survival of cancer cells. Upregulation of cellular DNA repair capacity is one of the most important cellular mechanisms that account for ineffective cervical anticancer radiotherapy and drug-based therapies.

Upregulation of cellular DNA repair capacity is one of the most important cellular mechanisms that account for ineffective cervical anticancer radiotherapy and drug-based therapies (Figure 3). According to Wagner group findings, incubating cells with lactate induces relaxation of chromatin, which translates into a transcriptionally active chromatin state and a subsequent positive regulation of essential genes implicated in cellular DNA repair, namely, *LIG4*, *NBS1*, and *APTX* (Wagner et al., 2015). Interestingly, impairment of DNA ligase IV (LIG4), nibrin (NBS1), or aprataxin (APTX) activity as a result of genes’ deleterious mutation leads to severe disorders in DNA damage response known as LIG4 syndrome, Nijmegen breakage syndrome (NBS), and Ataxia oculomotor apraxia-1 (Chun and Gatti, 2004; Chrzanowska et al., 2012; Altmann and Gennery, 2016). In the follow-up study with the use of selective ligand DHBA for lactate receptor, we have documented positive and significant effects of the lactate receptor stimulation on the mRNA expression of several genes including *BRCA1*, *BRCA2*, *NBS1*, *XRCC6*, and *PARD3*. The level of transcripts for *BRCA1* and *NBS1* was raised markedly and translated into a significantly higher protein level (BRCA1). In the complementary experiments with the use of shRNA, detrimental effects of HCAR1 silencing were observed on BRCA1 and NBS1 protein nuclear localization as their nuclear presence dropped markedly (Wagner et al., 2017b). Along with BRCA1 and nibrin, which represent crucial elements of the DNA homologue repair (HR) pathway, the key enzyme of the non-homologue end-joining pathway (NHEJ), namely, DNA-PKcs (DNA-dependent protein kinase catalytic subunit), has also been found to be profoundly affected by intracellular lactate presence. Recently, nuclear translocation of DNA-PKcs in cervical epithelial cancer cells has been observed after stimulation with L- or D-lactate. In particular, the enhanced presence of highly DNA-PKcs positive cells was reported for HeLa, CaSki, and C33A cell lines. Similarly, the lactate-driven DNA-PKcs translocation phenomenon was also found to be true for glioblastoma cells M059K (Wagner et al., 2021). Additionally, L- and D-lactate were also reported to induce activation of DNA-PKcs in nuclear compartment by phosphorylation of the protein at Ser 2056 (Wagner et al., 2015).

Another DNA repair gene regulated by L-lactate has been reported in the study by Govoni and colleagues (Govoni et al., 2021). Authors reported a decrease in cisplatin activity against gastrointestinal cancer cells, which was the consequence of upregulation of genes involved in DNA repair after exposition to L-lactate. These changes included enhanced expression of mismatch repair system (MMR) and nucleotide excision DNA repair genes, for example, *LIG1* and *PCNA*, which presumably diminished cisplatin antineoplastic potency. Conversely, inhibition of LDH activity, a lactate-limiting enzyme, downregulated expression of numerous genes of homologue recombination: *BRCA1*, *BRCA2*, *RAD50*, *RAD51*, *MRE11*, and *NBS1* in SW620 cells. Predictably, the effects of LDH inhibition were mostly reversed by supplementation of cancer cells with 10 mM L-lactate (Balboni et al., 2021). According to recent reports by Qu and colleagues, lactate originated from *Candida tropicalis*, a conditional pathogenic fungus in colorectal cancer patients, has been found to downregulate the expression of MLH1 involved in MMR through the HCA1-cAMP-PKA-CREB axis in colorectal cancer cells (Qu et al., 2021).

Collectively, such upregulation of numerous DNA repair proteins accounting for enhanced DNA repair mechanisms in a lactate-rich environment could inevitably translate into accelerated processing of DNA damage and resistance to DNA-damaging-based anticancer modalities.

## 5 The role of lactate in maintaining DNA protection through modulation of multidrug-resistance proteins

In physiological conditions, the ATP-binding cassette (ABC) protein family is responsible for preventing over-accumulation of toxins within the cell by active efflux of many xenobiotics, including anthracyclines, taxanes, and vinca alkaloids, from the cell. Many of them interact with DNA and cause its damage, resulting in cell death. While such a mechanism supporting cells is a desirable feature in normal cells, the efflux may protect tumor cells against anticancer drugs. Thus, their presence in many tumors often results in multidrug resistance, which is responsible for unsuccessful cancer therapy outcomes. One of the best studied transporters of the ABC protein family is ABCB1 (ATP-binding cassette sub-family B member 1; P-glycoprotein), known to render chemoresistance to several clinically used chemotherapeutics such as doxorubicin, paclitaxel, and vincristine (Yang et al., 2017). Other well-known protein-mediated multidrug-resistance phenotypes are ABCG2 (BRCP) and ABCC1 (MRP1), involved in chemoresistance to amptothecin analogues and vinca alkaloids, folate-based antimetabolites, anthracyclines, and anti-androgens (Moitra, 2015; Yang et al., 2017).

**FIGURE 4:**
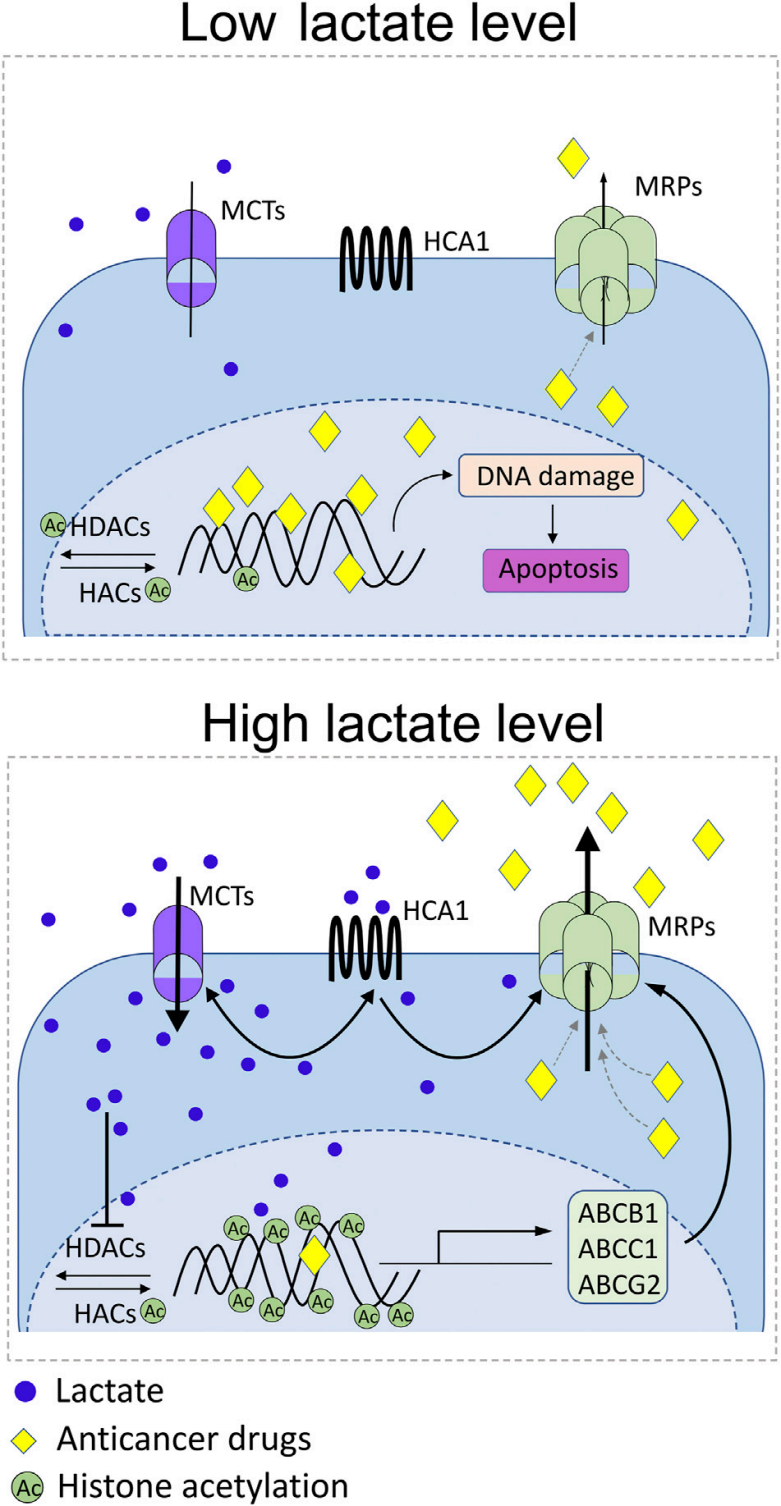
Schematic representation of the known reciprocal interplays between lactate receptor (HCA1), lactate transporters (MCTs), and multidrug-resistance proteins (MRPs). Under modest lactate levels, DNA-damaging anticancer drugs reach therapeutic concentration inside cancer cell, exert DNA damage, and trigger apoptosis (low lactate level). Rich lactate conditions in the tumor niche as a consequence of excessive glycolysis of cancer cells or symbiotic microbiota activity drive MCT-dependent lactate flux and develop epigenetic changes that positively drive MRP expression. Parallelly, lactate stimulates surface HCA1 that positively regulates expression of MCT1/4 and MRPs. Positive feedback loop of lactate-HCA1-MCT-lactate and lactate-HCA1-MRPs axis boosts activity of ABCB1, ABCC1, and ABCG2 in cancer cells along with decrease of anticancer drug bioavailability.

Interestingly, accumulating scientific evidence indicates the role of lactate and its metabolites in regulating the ABC proteins responsible for the induction of multidrug resistance (Figure 4). In our previous studies, we revealed upregulation of ABCB1 in lactate-stimulated cervical cancer cells in an HCA1-dependent manner (Wagner et al., 2017a). Indeed, HCA1 silencing resulted in downregulation of ABCB1, suggesting the presence of a direct relationship between both transmembrane proteins. In addition, diminished expression of ABCB1 in HCA1-compromised cells translated into a higher accumulation of doxorubicin in cells, while lactate receptor stimulation with DHBA, the HCA1 agonist, resulted in a lower accumulation of ABCB1 substrates in cells. In other words, cells exhibiting reduced expression of ABCB1 as a consequence of HCA1 deprivation showed pronounced sensitivity to growth inhibition after exposition to doxorubicin in comparison to control cells, while wild-type HeLa treated with DHBA exhibited acquired resistance to apoptosis after doxorubicin treatment. Convergent results have recently been observed in hepatic carcinoma cells, where lactate stimulation resulted in an ABCB1-dependent induction of chemoresistance (Soni et al., 2020). Soni and colleagues showed lactate-induced chemoresistance against doxorubicin along with upregulation of HCA1 protein and increased expression of the ABCB1 gene. Similar to our observation, downregulation of HCA1 sensitized cells to doxorubicin treatment, which was correlated with ABCB1 downregulation (Soni et al., 2020). Taking into account the abovementioned observations, we postulate that lactate modulates the level of ABCB1 protein in an HCA1-dependent manner. Nevertheless, it seems that lactate might also be involved in regulating ABC transporters in the HCA1 independent pathway. Previously, lactate was reported to induce Snail expression through the TGF-β1-dependent pathway (Li et al., 2018) and induce TAZ expression in lung cancer cells (Huang et al., 2020). Later, Dong and colleagues proposed that Snail and TAZ coordinated by lactate form complexes with AP-1 which, in turn, activate ABCC1 expression in non-small cell lung cancer cells (Dong et al., 2020). In particular, they have shown resistance of lung cancer cells to etoposide as a result of lactate-induced ABCC1 expression. Finally, it seems that lactate might also regulate ABC transporters’ expression more directly through epigenetic mechanisms. It has been proposed that lactate may be an important transcriptional regulator through inhibition of HDAC activity and induction of histone hyperacetylation, both known for deregulation of gene transcription (Latham et al., 2012). Moreover, it is well established that the expression and function of ABC proteins like ABCB1, ABCC1, and ABCG2 are modulated by pharmacological inhibition of HDACs (Shi et al., 2020; You et al., 2020). Hence, it clearly appears that lactate can also modulate the level of ABC transporters by regulating the level of histone acetylation.

## 6 Lactate and short-chain fatty acids link host DNA repair mechanisms and chromatin rearrangement with viral life cycle

The cellular DNA repair machinery is responsible for protection of the host genome integrity by responding to damaged nuclear DNA or sensing mis-localized self-nucleic acids. After viral infection or during viral replication, foreign genetic material is spread in different intracellular compartments. Such viral nucleic acid forms can imitate unrepaired host DNA in the nucleus, thus recruiting and activating canonical downstream signaling pathways of cellular DSB (DNA double-strand break) repair machinery. Since DSB repair systems, mostly HR and NHEJ, have the ability to sense foreign or damaged nucleic acids, their cellular components may have extensive interactions with numerous different viruses (Hristova et al., 2020). Such mutual interactions can restrict viral infection into the cell by signaling its invasion and/or hamper virus replication and growth by restraining viral nucleic acids. These observations are evident for numerous NHEJ components and viral counterparts, for example, herpes simplex virus 1 (HSV-1) (Trigg et al., 2017), Kaposi sarcoma-associated herpesvirus (KSHV) (Cha et al., 2010), vaccinia virus (VACV) (Peters et al., 2013), human T-cell leukemia virus type (HTLV-1) (Wang et al., 2017), hepatitis B virus (HBV) (Li et al., 2016), and more. Opposed proviral activities of NHEJ were also observed as a consequence of neutralization or hijacking of NHEJ components by viral processes for their own benefit. In particular, such interactions that lead to activation or enhancement of the viral life cycle have been reported for adenoviruses (AV) (Baker et al., 2007; Nebenzahl-Sharon et al., 2019), adeno-associated viruses (AAV) (Choi et al., 2010), herpes simplex virus 1 (Muylaert and Elias, 2007; Smith et al., 2014; Edwards et al., 2018), VACV (Luteijn et al., 2018), John Cunningham virus (JC) (Darbinyan et al., 2004), and parvovirus B19 (Munakata et al., 2005), among others. Interestingly, the indispensable role of the NHEJ repair system in retroviral integration is widely accepted, though the role of DNA-PKcs in the HIV cycle is still disputable, especially in non-CD4 cells (Liu et al., 2013; Hughes et al., 2020; Wagner et al., 2021). The presence of viral proteins and their activity in host cells often contribute to altered activity of DSB repair pathways together with the attraction of particular DNA repair proteins to viral replication sites. Apart from NHEJ, HR repair (Mehta and Laimins, 2018), base excision repair (BER) (Bogani et al., 2009), nucleotide excision repair (NER) (Lu et al., 2007; Feng et al., 2019), single-strand break (SSB) repair (Nukuzuma et al., 2013) and mismatch repair (MMR) systems (Wang et al., 2008) could also serve as the interaction site with a range of proteins of viral origin.

Symbiotic microbiota colonization of the skin and mucosal surfaces is fundamental for human life. Mucosal microorganisms generate a range of biologically active molecules that not only protect against pathogen colonization but also regulate the host defense response against infectious pathogens (Pickard et al., 2017). The primary end products resulting from intestinal microbes’ carbohydrates and starch catabolism are short-chain fatty acids: acetate, butyrate, and propionate, among others. L- and D-Lactate produced by most mucosal microbiota can be rapidly utilized by converting these molecules to butyrate (Bourriaud et al., 2005). Butyrate has received great attention since first observations on its effects on reactivation of several viruses *in vitro* and *in vivo* and possible use as a therapeutic arm against the virus-associated malignancies. Such a mechanism of butyrate-mediated regulation of the viral latent-to-lytic switch must involve a complex model of chromatin reshaping. This most likely includes mechanisms of histone modifications and acetylation of nonhistone proteins through HDAC inhibition and possibly recruitment of host DNA repair proteins. Westphal and colleagues showed that clinical doses of γ-irradiation and butyrate can induce lytic infection of EBV in lymphoblastoid cell lines as well as in EBV-positive B-cell tumors *in vivo* (Westphal et al., 2000). Similarly, butyrate treatment of latently infected B-cells with EBV resulted in the induction of EBV lytic-phase genes and gene products (thymidine kinase) and driven apoptosis in cells concomitantly treated with ganciclovir (Perrine et al., 2007). Among other SCFA small molecules, phenylbutyrate and valproic acid have also been studied as oncolytic primers in cells, mice, and patients harboring EBV or KSHV (Feng and Kenney, 2006; Phillips and Griffin, 2007; Lechowicz et al., 2009; Jones et al., 2010). Additionally, multiple SCFAs isolated from periodontal pathogens have been shown to reactivate retroviruses in Jurkat and primary T-cell models of HIV-1 latency (Das et al., 2015). In these experiments, the authors observed higher concentrations of butyric acid, isobutyric acid, isovaleric acid, propionic acid, and acetic acid in the saliva of individuals suffering from periodontal disease. Concomitantly, the saliva of these patients strongly induced HIV-1 transcription through concurrent repression of histone methylation and acetylation at the proviral promoter.

Among other HDACs, special attention has been drawn to SIRT1 activity, which is implicated in cellular DNA repair processes and the viral life cycle (Budayeva et al., 2015). SIRT1 is a protein that belongs to a group of sirtuins (HDAC III) actively promoting HR in human cells, and this activity requires WRN (Werner helicase) (Uhl et al., 2010). According to a recent report by Taniguchi and colleagues, SIRT1 is a protein deacetylase involved in the maintenance of genome integrity by stabilizing the extrachromosomal amplification structures (Taniguchi et al., 2021). Thus, it is likely that the activity of cellular SIRT1 may be crucial for viral episome fate. Indeed, numerous studies have reported the involvement of SIRT1 in the regulation of HPV replication (Uhl et al., 2010; Das et al., 2017; James et al., 2020). Das and colleagues demonstrated that SIRT1 regulates the recruitment of the DNA repair protein WRN to the replicating DNA to control the levels and fidelity of replicating HPV 16 E1-E2 regulatory genes (Das et al., 2019). Furthermore, SIRT1 activity *via* acetylation of histones H1 (Lys26) and H4 (Lys16) supported both RAD51 and NBS1 recruitment to the HPV31 genome, thus demonstrating that SIRT1 can be a crucial controller of numerous facets of human papillomavirus life cycle (Langsfeld et al., 2015). In the HIV-1 life cycle, SIRT1 contributes directly to viral latency, and suppression of SIRT1 with Sirtinol (SIRT1 inhibitor) or SAHA induces HIV-1 transactivation (Das et al., 2015). Other SIRT1 inhibitors, nicotinamide and Ex257, suppressed HBV replication or transcription *in vitro* and *in vivo* (Li et al., 2016; Deng et al., 2017). Interestingly, inhibition of SIRT2 with the specific inhibitor AGK2 brought a new therapeutic option for controlling HBV infection (Yu et al., 2018). Recent literature shows a lack of direct evidence for lactate-mediated SIRT1 regulatory action on the viral life cycle. Nevertheless, lactate inhibitory effects on SIRT1 expression in kidney cells resulting in increased global H3 and H3K9 acetylation have been recently reported (Miranda-Gonçalves et al., 2020).

**FIGURE 5:**
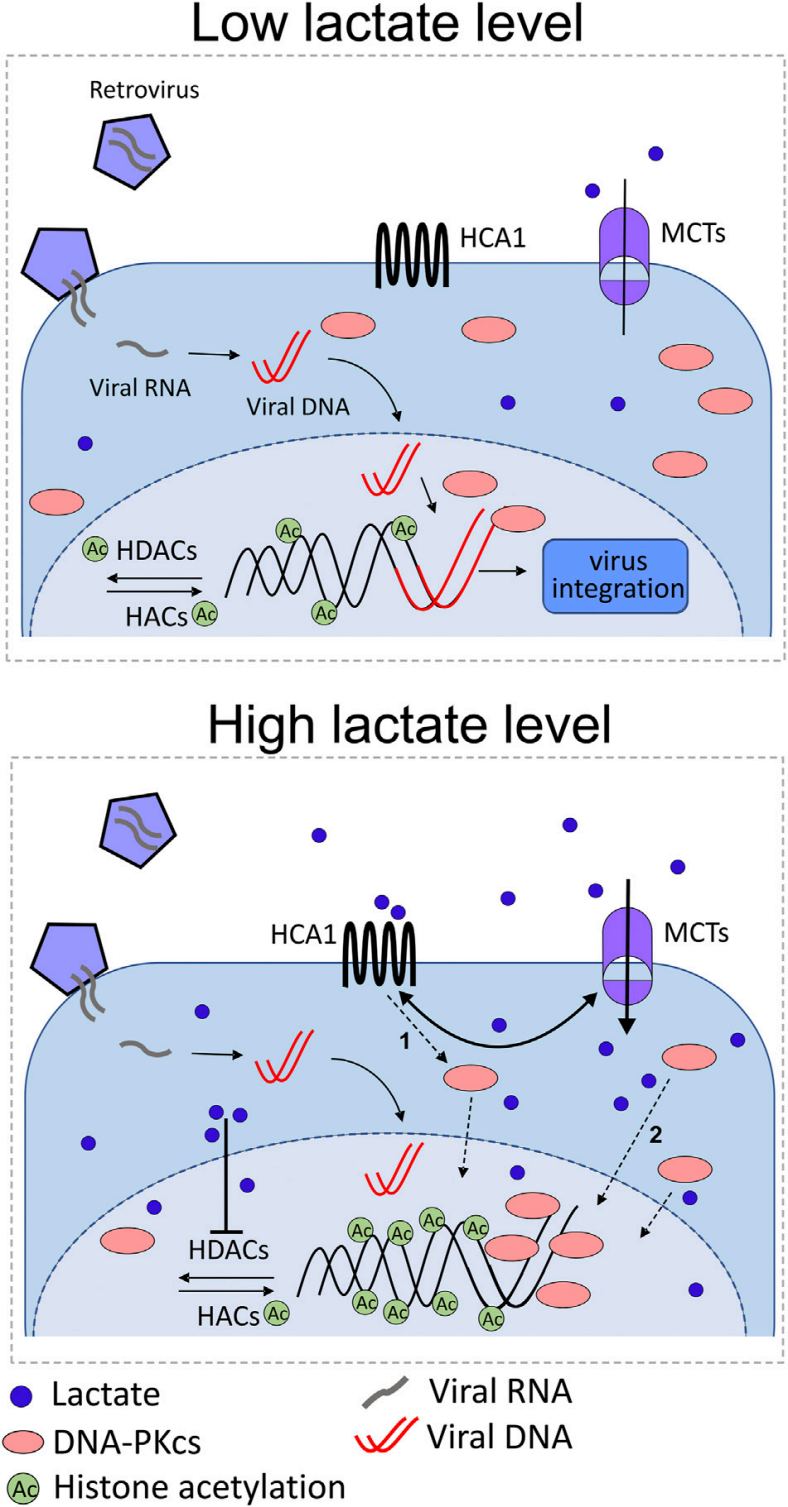
Illustration of the possible role of lactate in anti-retroviral protection of the female genital tract as a consequence of lactate action through its receptor (HCA1) and as a HDAC inhibitor. Under normal conditions (high lactate level), symbiotic microbiota produce L-/D-lactate which can be sensed by cervical epithelial cells equipped with a specific receptor for lactate. Microbiota-derived lactate stimulates surface HCA1, induces cAMP signaling, and triggers cAMP/EPAC/PKA-dependent shuttling of DNA-PKcs to nuclear compartment (1 pathway). HCA1 signaling also increases MCT expression followed by monocarboxylate influx, which, in turn, positively regulates HCA1 expression. In the nuclear compartment, lactate and butyrate inhibit histone deacetylases (HDACs), thus promoting histone acetylation by histone acetylases (HACs). Acetylated histones induce chromatin relaxation and recruitment of DNA-PKcs to the nucleus (2 pathways). Finally, enhanced nuclear localization of DNA-PKcs protects cells from lentiviral (e.g., HIV-1) transduction. Lactate shortage (low lactate level), for example, as a result of cervicovaginal microbiota dysbiosis, accounts for insufficient anti-lentiviral protection.

The recent study by Wagner and colleagues provided a new aspect of the host cells and commensal microbiota interplay in the female reproductive tract and demonstrated a distinctive mechanism implicated in the resistance of eukaryotic cells to viruses (Wagner et al., 2021). The high concentration of lactate maintained by symbiotic lactic acid bacteria is the exclusive feature of the lower female genital tract. This study has demonstrated, for the first time, that incubation of cervical cancer cells in the presence of lactate impairs the process of retrovirus transduction (Figure 5). As previously stated, lactate–cell stimulatory pathways comprise the activation of the membrane lactate-specific HCA1 receptor and the inhibition of HDAC that results in enhanced histone H3 and H4 acetylation and the decrease of nuclear chromatin compactness. In our experiments, we have reported a nuclear increase of DNA-PKcs in cervical epithelial cell cultures exposed to 20 mM of L- or D-lactate, concurrent with a reduction in transduction efficacy of retroviral vectors. In other words, DNA-PKcs translocation, which is the crucial protein of the NHEJ machinery translates into inhibition of lentiviral transduction rates. Though the DNA-PK complex was previously reported to be essential for efficient lentiviral integration, we have observed DNA-PKcs acting as a negative regulator of viral transduction. Probably, in this particular experimental setting, mitigated retroviral transduction success was a consequence of the unfavorable composition of the DNA–PK complex (DNA-PKcs, Ku70, and Ku80) (Anisenko et al., 2020) at the viral cDNA integration site. Further detailed experiments with the use of 3,5-dihydroxybenzoic acid, specific HCA1 agonist, and sodium butyrate, a potent HDAC inhibitor, demonstrated the combined two pathway-dependent effects of lactate on nuclear recruitment of DNA-PKcs. Taken together, these findings demonstrated the unique function of lactate at the ground of microorganism–mammalian interactions and illustrated the mechanism of the innate activity of lactate in the suppression of lentiviral transduction through elevated nuclear retention of DNA-PKcs.

## 7 Concluding remarks

Current research has demonstrated the dual role played by lactate in the cancer niche and lactate-rich microenvironment. Under physiological conditions, a high lactate level reproduces highly accessible nuclear environment for DNA repair mechanisms, thus stimulating DNA repair dynamics, significantly enhancing wound healing, and suppressing retrovirus infection. On the other hand, malignant tissue benefits from lactate presence to increase its resistance to anticancer therapies based on drug and ionizing radiation-targeting cancer DNA damage. The theatre of mutual interactions gets even more complex in the zone of influence of commensal microbiota. Lactic acid bacteria are constantly shaped by age, diet, and whole-life exposures to drugs that also affect their metabolite production and thus possible lactate and SCFA interaction capacity. Collectively, lactate represents a small molecule with a significant impact on local and distant homeostasis with the feature of a double-edged sword in human life.

## References

[B1] Ahmed K. (2011). Biological roles and therapeutic potential of hydroxy-carboxylic Acid receptors. Front. Endocrinol. 2, 51. 10.3389/fendo.2011.00051 PMC3356039 22654812

[B2] Allaman I. Bélanger M. Magistretti P. J. (2015). Methylglyoxal, the dark side of glycolysis. Front. Neurosci. 9, 23. 10.3389/fnins.2015.00023 25709564 PMC4321437

[B3] Altmann T. Gennery A. R. (2016). DNA ligase IV syndrome; a review. Orphanet J. Rare Dis. 11 (1), 137. 10.1186/s13023-016-0520-1 27717373 PMC5055698

[B4] Amabebe E. Anumba D. (2020). Female gut and genital tract microbiota-induced crosstalk and differential effects of short-chain fatty acids on immune sequelae. Front. Immunol. 11, 2184. 10.3389/fimmu.2020.02184 33013918 PMC7511578

[B5] Anisenko A. Kan M. Shadrina O. Brattseva A. Gottikh M. (2020). Phosphorylation targets of DNA-PK and their role in HIV-1 replication. Cells 9 (8), 1907. 10.3390/cells9081907 PMC7464883 32824372

[B6] Baker A. Rohleder K. J. Hanakahi L. A. Ketner G. (2007). Adenovirus E4 34k and E1b 55k oncoproteins target host DNA ligase IV for proteasomal degradation. J. Virol. 81 (13), 7034–7040. 10.1128/JVI.00029-07 17459921 PMC1933317

[B7] Balboni A. Govoni M. Rossi V. Roberti M. Cavalli A. Di Stefano G. (2021). Lactate dehydrogenase inhibition affects homologous recombination repair independently of cell metabolic asset; implications for anticancer treatment. Biochim. Biophys. Acta. Gen. Subj. 1865 (1), 129760. 10.1016/j.bbagen.2020.129760 33035602

[B8] Belenguer A. Duncan S. H. Holtrop G. Anderson S. E. Lobley G. E. Flint H. J. (2007). Impact of pH on lactate formation and utilization by human fecal microbial communities. Appl. Environ. Microbiol. 73 (20), 6526–6533. 10.1128/AEM.00508-07 17766450 PMC2075063

[B9] Bhatt A. N. Chauhan A. Khanna S. Rai Y. Singh S. Soni R. (2015). Transient elevation of glycolysis confers radio-resistance by facilitating DNA repair in cells. BMC Cancer 15, 335. 10.1186/s12885-015-1368-9 25925410 PMC4425929

[B10] Bogani F. Chua C. N. Boehmer P. E. (2009). Reconstitution of uracil DNA glycosylase-initiated base excision repair in herpes simplex virus-1. J. Biol. Chem. 284 (25), 16784–16790. 10.1074/jbc.M109.010413 19411250 PMC2719314

[B11] Bourriaud C. Robins R. J. Martin L. Kozlowski F. Tenailleau E. Cherbut C. (2005). Lactate is mainly fermented to butyrate by human intestinal microfloras but inter-individual variation is evident. J. Appl. Microbiol. 99 (1), 201–212. 10.1111/j.1365-2672.2005.02605.x 15960680

[B12] Bozzo L. Puyal J. Chatton J. Y. (2013). Lactate modulates the activity of primary cortical neurons through a receptor-mediated pathway. PloS One 8 (8), e71721. 10.1371/journal.pone.0071721 23951229 PMC3741165

[B13] Brown T. P. Ganapathy V. (2020). Lactate/GPR81 signaling and proton motive force in cancer: Role in angiogenesis, immune escape, nutrition, and Warburg phenomenon. Pharmacol. Ther. 206, 107451. 10.1016/j.pharmthera.2019.107451 31836453

[B14] Budayeva H. G. Rowland E. A. Cristea I. M. (2015). Intricate roles of mammalian sirtuins in defense against viral pathogens. J. Virol. 90 (1), 5–8. 10.1128/JVI.03220-14 26491165 PMC4702534

[B15] Carretta M. D. Quiroga J. López R. Hidalgo M. A. Burgos R. A. (2021). Participation of short-chain fatty acids and their receptors in gut inflammation and colon cancer. Front. Physiol. 12, 662739. 10.3389/fphys.2021.662739 33897470 PMC8060628

[B16] Certo M. Tsai C. H. Pucino V. Ho P. C. Mauro C. (2021). Lactate modulation of immune responses in inflammatory versus tumour microenvironments. Nat. Rev. Immunol. 21 (3), 151–161. 10.1038/s41577-020-0406-2 32839570

[B17] Cha S. Lim C. Lee J. Y. Song Y. J. Park J. Choe J. (2010). DNA-PK/Ku complex binds to latency-associated nuclear antigen and negatively regulates Kaposi's sarcoma-associated herpesvirus latent replication. Biochem. Biophys. Res. Commun. 394 (4), 934–939. 10.1016/j.bbrc.2010.03.086 20303334

[B18] Chen A. N. Luo Y. Yang Y. H. Fu J. T. Geng X. M. Shi J. P. (2021). Lactylation, a novel metabolic reprogramming code: Current status and prospects. Front. Immunol. 12, 688910. 10.3389/fimmu.2021.688910 34177945 PMC8222712

[B19] Chen J. Ding Z. Peng Y. Pan F. Li J. Zou L. (2014). HIF-1α inhibition reverses multidrug resistance in colon cancer cells via downregulation of MDR1/P-glycoprotein. PloS one 9 (6), e98882. 10.1371/journal.pone.0098882 24901645 PMC4047061

[B20] Cheng G. M. To K. K. (2012). Adverse cell culture conditions mimicking the tumor microenvironment upregulate ABCG2 to mediate multidrug resistance and a more malignant phenotype. ISRN Oncol. 2012, 746025. 10.5402/2012/746025 22778999 PMC3384895

[B21] Choi Y. K. Nash K. Byrne B. J. Muzyczka N. Song S. (2010). The effect of DNA-dependent protein kinase on adeno-associated virus replication. PloS One 5 (12), e15073. 10.1371/journal.pone.0015073 21188139 PMC3004791

[B22] Chrzanowska K. H. Gregorek H. Dembowska-Bagińska B. Kalina M. A. Digweed M. (2012). Nijmegen breakage syndrome (NBS). Orphanet J. Rare Dis. 7, 13. 10.1186/1750-1172-7-13 22373003 PMC3314554

[B23] Chun H. H. Gatti R. A. (2004). Ataxia-telangiectasia, an evolving phenotype. DNA Repair 3 (8-9), 1187–1196. 10.1016/j.dnarep.2004.04.010 15279807

[B24] Colegio O. R. Chu N. Q. Szabo A. L. Chu T. Rhebergen A. M. Jairam V. (2014). Functional polarization of tumour-associated macrophages by tumour-derived lactic acid. Nature 513 (7519), 559–563. 10.1038/nature13490 25043024 PMC4301845

[B25] Darbinyan A. Siddiqui K. M. Slonina D. Darbinian N. Amini S. White M. K. (2004). Role of JC virus agnoprotein in DNA repair. J. Virol. 78 (16), 8593–8600. 10.1128/JVI.78.16.8593-8600.2004 15280468 PMC479055

[B26] Das B. Dobrowolski C. Shahir A. M. Feng Z. Yu X. Sha J. (2015). Short chain fatty acids potently induce latent HIV-1 in T-cells by activating P-TEFb and multiple histone modifications. Virology 474, 65–81. 10.1016/j.virol.2014.10.033 25463605 PMC4259832

[B27] Das D. Bristol M. L. Smith N. W. James C. D. Wang X. Pichierri P. (2019). Werner helicase control of human papillomavirus 16 E1-E2 DNA replication is regulated by SIRT1 deacetylation. mBio 10 (2), 002633–e319. 10.1128/mBio.00263-19 PMC6426601 30890607

[B28] Das D. Smith N. Wang X. Morgan I. M. (2017). The deacetylase SIRT1 regulates the replication properties of human papillomavirus 16 E1 and E2. J. Virol. 91 (10), e00102–e00117. 10.1128/JVI.00102-17 28275188 PMC5411580

[B29] DeBerardinis R. J. Mancuso A. Daikhin E. Nissim I. Yudkoff M. Wehrli S. (2007). Beyond aerobic glycolysis: Transformed cells can engage in glutamine metabolism that exceeds the requirement for protein and nucleotide synthesis. Proc. Natl. Acad. Sci. U. S. A. 104 (49), 19345–19350. 10.1073/pnas.0709747104 18032601 PMC2148292

[B30] Deng J. J. Kong K. E. Gao W. W. Tang H. V. Chaudhary V. Cheng Y. (2017). Interplay between SIRT1 and Hepatitis B virus X protein in the activation of viral transcription. Biochim. Biophys. Acta. Gene Regul. Mech. 1860 (4), 491–501. 10.1016/j.bbagrm.2017.02.007 28242208

[B31] Dienel G. A. (2019). Brain glucose metabolism: Integration of energetics with function. Physiol. Rev. 99 (1), 949–1045. 10.1152/physrev.00062.2017 30565508

[B32] Dodd A. L. Yang J. Shen M. H. Sampson J. R. Tee A. R. (2015). mTORC1 drives HIF-1α and VEGF-A signalling via multiple mechanisms involving 4E-BP1, S6K1 and STAT3. Oncogene 34 (17), 2239–2250. 10.1038/onc.2014.164 24931163 PMC4172452

[B33] Doherty J. R. Yang C. Scott K. E. Cameron M. D. Fallahi M. Li W. (2014). Blocking lactate export by inhibiting the Myc target MCT1 Disables glycolysis and glutathione synthesis. Cancer Res. 74 (3), 908–920. 10.1158/0008-5472.CAN-13-2034 24285728 PMC3946415

[B34] Domingue P. A. Sadhu K. Costerton J. W. Bartlett K. Chow A. W. (1991). The human vagina: Normal flora considered as an *in situ* tissue-associated, adherent biofilm. Genitourin. Med. 67 (3), 226–231. 10.1136/sti.67.3.226 2071125 PMC1194677

[B35] Dong Q. Zhou C. Ren H. Zhang Z. Cheng F. Xiong Z. (2020). Lactate-induced MRP1 expression contributes to metabolism-based etoposide resistance in non-small cell lung cancer cells. Cell Commun. Signal. 18 (1), 167. 10.1186/s12964-020-00653-3 33097055 PMC7583203

[B36] Duncan S. H. Barcenilla A. Stewart C. S. Pryde S. E. Flint H. J. (2002). Acetate utilization and butyryl coenzyme A (CoA):acetate-CoA transferase in butyrate-producing bacteria from the human large intestine. Appl. Environ. Microbiol. 68 (10), 5186–5190. 10.1128/AEM.68.10.5186-5190.2002 12324374 PMC126392

[B37] Edwards T. G. Bloom D. C. Fisher C. (2018). The ATM and rad3-related (ATR) protein kinase pathway is activated by herpes simplex virus 1 and required for efficient viral replication. J. Virol. 92 (6), 018844–e1917. 10.1128/JVI.01884-17 PMC5827400 29263259

[B38] Englander H. R. Shklair I. L. Fosdick L. S. (1959). Shklair IL, fosdick ls: The effects of saliva on the pH and lactate concentration in dental plaques. I. Caries-rampant individuals. J. Dent. Res. 38, 848–853. 10.1177/00220345590380051201 13820374

[B39] Feng J. Yang G. Liu Y. Gao Y. Zhao M. Bu Y. (2019). LncRNA PCNAP1 modulates Hepatitis B virus replication and enhances tumor growth of liver cancer. Theranostics 9 (18), 5227–5245. 10.7150/thno.34273 31410212 PMC6691589

[B40] Feng J. Yang H. Zhang Y. Wei H. Zhu Z. Zhu B. (2017). Tumor cell-derived lactate induces TAZ-dependent upregulation of PD-L1 through GPR81 in human lung cancer cells. Oncogene 36 (42), 5829–5839. 10.1038/onc.2017.188 28604752

[B41] Feng W. H. Kenney S. C. (2006). Valproic acid enhances the efficacy of chemotherapy in EBV-positive tumors by increasing lytic viral gene expression. Cancer Res. 66 (17), 8762–8769. 10.1158/0008-5472.CAN-06-1006 16951192

[B42] Fiaschi T. Marini A. Giannoni E. Taddei M. L. Gandellini P. De Donatis A. (2012). Reciprocal metabolic reprogramming through lactate shuttle coordinately influences tumor-stroma interplay. Cancer Res. 72 (19), 5130–5140. 10.1158/0008-5472.CAN-12-1949 22850421

[B43] Goodwin M. L. Gladden L. B. Nijsten M. W. Jones K. B. (2015). Lactate and cancer: Revisiting the warburg effect in an era of lactate shuttling. Front. Nutr. 1, 27. 10.3389/fnut.2014.00027 25988127 PMC4428352

[B44] Gottfried E. Kunz-Schughart L. A. Ebner S. Mueller-Klieser W. Hoves S. Andreesen R. (2006). Tumor-derived lactic acid modulates dendritic cell activation and antigen expression. Blood 107 (5), 2013–2021. 10.1182/blood-2005-05-1795 16278308

[B45] Govoni M. Rossi V. Di Stefano G. Manerba M. (2021). Lactate upregulates the expression of DNA repair genes, causing intrinsic resistance of cancer cells to cisplatin. Pathol. Oncol. Res. 27, 1609951. 10.3389/pore.2021.1609951 34987311 PMC8720744

[B46] Harachi M. Masui K. Okamura Y. Tsukui R. Mischel P. S. Shibata N. (2018). mTOR complexes as a nutrient sensor for driving cancer progression. Int. J. Mol. Sci. 19 (10), 3267. 10.3390/ijms19103267 PMC6214109 30347859

[B47] Hielscher A. Qiu C. Porterfield J. Smith Q. Gerecht S. (2013). Hypoxia affects the structure of breast cancer cell-derived matrix to support angiogenic responses of endothelial cells. J. Carcinog. Mutagen. 13, 005. 10.4172/2157-2518.S13-005 24600535 PMC3940068

[B48] Hirschhaeuser F. Sattler U. G. Mueller-Klieser W. (2011). Lactate: A metabolic key player in cancer. Cancer Res. 71 (22), 6921–6925. 10.1158/0008-5472.CAN-11-1457 22084445

[B49] Hoque R. Farooq A. Ghani A. Gorelick F. Mehal W. Z. (2014). Lactate reduces liver and pancreatic injury in Toll-like receptor- and inflammasome-mediated inflammation via GPR81-mediated suppression of innate immunity. Gastroenterology 146 (7), 1763–1774. 10.1053/j.gastro.2014.03.014 24657625 PMC4104305

[B50] Hristova D. B. Lauer K. B. Ferguson B. J. (2020). Viral interactions with non-homologous end-joining: A game of hide-and-seek. J. Gen. Virol. 101 (11), 1133–1144. 10.1099/jgv.0.001478 32735206 PMC7879558

[B51] Huang T. Zhou X. Mao X. Yu C. Zhang Z. Yang J. (2020). Lactate-fueled oxidative metabolism drives DNA methyltransferase 1-mediated transcriptional co-activator with PDZ binding domain protein activation. Cancer Sci. 111 (1), 186–199. 10.1111/cas.14246 31746077 PMC6942427

[B52] Hughes K. Akturk G. Gnjatic S. Chen B. Klotman M. Blasi M. (2020). Proliferation of HIV-infected renal epithelial cells following virus acquisition from infected macrophages. AIDS 34 (11), 1581–1591. 10.1097/QAD.0000000000002589 32701578 PMC7579771

[B53] Ishihara S. Hata K. Hirose K. Okui T. Toyosawa S. Uzawa N. (2022). The lactate sensor GPR81 regulates glycolysis and tumor growth of breast cancer. Sci. Rep. 12 (1), 6261. 10.1038/s41598-022-10143-w 35428832 PMC9012857

[B54] James C. D. Das D. Morgan E. L. Otoa R. Macdonald A. Morgan I. M. (2020). Werner syndrome protein (WRN) regulates cell proliferation and the human papillomavirus 16 life cycle during epithelial differentiation. mSphere 5 (5), 008588–e920. 10.1128/mSphere.00858-20 PMC7494838 32938703

[B55] Jia Q. Xu O. Wang J. Dong J. Ren X. Jia X. (2020). Effects of GPR81 silencing combined with cisplatin stimulation on biological function in hypopharyngeal squamous cell carcinoma. Mol. Med. Rep. 22 (3), 1727–1736. 10.3892/mmr.2020.11255 32582969 PMC7411294

[B56] Jones K. Nourse J. Corbett G. Gandhi M. K. (2010). Sodium valproate in combination with ganciclovir induces lysis of EBV-infected lymphoma cells without impairing EBV-specific T-cell immunity. Int. J. Lab. Hematol. 32 (1), e169–e174. 10.1111/j.1751-553X.2008.01130.x 19196381

[B57] Kai A. K. Chan L. K. Lo R. C. Lee J. M. Wong C. C. Wong J. C. (2016). Down-regulation of TIMP2 by HIF-1α/miR-210/HIF-3α regulatory feedback circuit enhances cancer metastasis in hepatocellular carcinoma. Hepatology 64 (2), 473–487. 10.1002/hep.28577 27018975 PMC5074303

[B58] Kalluri R. (2016). The biology and function of fibroblasts in cancer. Nat. Rev. Cancer 16 (9), 582–598. 10.1038/nrc.2016.73 27550820

[B59] Koukourakis M. I. Giatromanolaki A. Sivridis E. Bougioukas G. Didilis V. Gatter K. C. (2003). Lactate dehydrogenase-5 (LDH-5) overexpression in non-small-cell lung cancer tissues is linked to tumour hypoxia, angiogenic factor production and poor prognosis. Br. J. Cancer 89, 877–885. 10.1038/sj.bjc.6601205 12942121 PMC2394471

[B60] Langsfeld E. S. Bodily J. M. Laimins L. A. (2015). The deacetylase sirtuin 1 regulates human papillomavirus replication by modulating histone acetylation and recruitment of DNA damage factors NBS1 and Rad51 to viral genomes. PLoS Pathog. 11 (9), e1005181. 10.1371/journal.ppat.1005181 26405826 PMC4583417

[B61] Latham T. Mackay L. Sproul D. Karim M. Culley J. Harrison D. J. (2012). Lactate, a product of glycolytic metabolism, inhibits histone deacetylase activity and promotes changes in gene expression. Nucleic Acids Res. 40 (11), 4794–4803. 10.1093/nar/gks066 22323521 PMC3367171

[B62] Lechowicz M. Dittmer D. P. Lee J. Y. Krown S. E. Wachsman W. Aboulafia D. (2009). Molecular and clinical assessment in the treatment of AIDS Kaposi sarcoma with valproic Acid. Clin. Infect. Dis. 49 (12), 1946–1949. 10.1086/648447 19911999 PMC2952388

[B63] Lee Y. J. Shin K. J. Park S. A. Park K. S. Park S. Heo K. (2016). G-protein-coupled receptor 81 promotes a malignant phenotype in breast cancer through angiogenic factor secretion. Oncotarget 7 (43), 70898–70911. 10.18632/oncotarget.12286 27765922 PMC5342597

[B64] Lee Y. S. Kim T. Y. Kim Y. Lee S. H. Kim S. Kang S. W. (2018). Microbiota-derived lactate accelerates intestinal stem-cell-mediated epithelial development. Cell Host Microbe 24 (6), 833–846. 10.1016/j.chom.2018.11.002 30543778

[B65] Li G. Wang H. Q. Wang L. H. Chen R. P. Liu J. P. (2014). Distinct pathways of ERK1/2 activation by hydroxy-carboxylic acid receptor-1. PloS One 9 (3), e93041. 10.1371/journal.pone.0093041 24671202 PMC3966839

[B66] Li W. Y. Ren J. H. Tao N. N. Ran L. K. Chen X. Zhou H. Z. (2016a). The SIRT1 inhibitor, nicotinamide, inhibits Hepatitis B virus replication *in vitro* and *in vivo* . Arch. Virol. 161 (3), 621–630. 10.1007/s00705-015-2712-8 26660162

[B67] Li X. Zhang Z. Zhang Y. Cao Y. Wei H. Wu Z. (2018). Upregulation of lactate-inducible snail protein suppresses oncogene-mediated senescence through p16^INK4a^ inactivation. J. Exp. Clin. Cancer Res. 37 (1), 39. 10.1186/s13046-018-0701-y 29482580 PMC5828408

[B68] Li Y. Sun X. X. Qian D. Z. Dai M. S. (2020). Molecular crosstalk between MYC and HIF in cancer. Front. Cell Dev. Biol. 8, 590576. 10.3389/fcell.2020.590576 33251216 PMC7676913

[B69] Li Y. Wu Y. Zheng X. Cong J. Liu Y. Li J. (2016b). Cytoplasm-translocated ku70/80 complex sensing of HBV DNA induces hepatitis-associated chemokine secretion. Front. Immunol. 7, 569. 10.3389/fimmu.2016.00569 27994596 PMC5136554

[B70] Liberti M. V. Locasale J. W. (2016). The warburg effect: How does it benefit cancer cells? Trends biochem. Sci. 41 (3), 211–218. 10.1016/j.tibs.2015.12.001 26778478 PMC4783224

[B71] Liu C. Wu J. Zhu J. Kuei C. Yu J. Shelton J. (2009). Lactate inhibits lipolysis in fat cells through activation of an orphan G-protein-coupled receptor, GPR81. J. Biol. Chem. 284 (5), 2811–2822. 10.1074/jbc.M806409200 19047060

[B72] Liu P. Ge M. Hu J. Li X. Che L. Sun K. (2017). A functional mammalian target of rapamycin complex 1 signaling is indispensable for c-Myc-driven hepatocarcinogenesis. Hepatology 66 (1), 167–181. 10.1002/hep.29183 28370287 PMC5481473

[B73] Liu R. Huang L. Li J. Zhou X. Zhang H. Zhang T. (2013). HIV Infection in gastric epithelial cells. J. Infect. Dis. 208 (8), 1221–1230. 10.1093/infdis/jit314 23852124

[B74] Liu Y. An S. Ward R. Yang Y. Guo X. X. Li W. (2016). G protein-coupled receptors as promising cancer targets. Cancer Lett. 376 (2), 226–239. 10.1016/j.canlet.2016.03.031 27000991

[B75] Lu C. C. Chen Y. C. Wang J. T. Yang P. W. Chen M. R. (2007). Xeroderma pigmentosum C is involved in Epstein Barr virus DNA replication. J. Gen. Virol. 88 (12), 3234–3243. 10.1099/vir.0.83212-0 18024891

[B76] Luteijn R. D. Drexler I. Smith G. L. Lebbink R. J. Wiertz E. (2018). Mutagenic repair of double-stranded DNA breaks in vaccinia virus genomes requires cellular DNA ligase IV activity in the cytosol. J. Gen. Virol. 99 (6), 790–804. 10.1099/jgv.0.001034 29676720 PMC7614823

[B77] Lv Y. Zhao S. Han J. Zheng L. Yang Z. Zhao L. (2015). Hypoxia-inducible factor-1α induces multidrug resistance protein in colon cancer. Onco. Targets. Ther. 8, 1941–1948. 10.2147/OTT.S82835 26251616 PMC4524588

[B78] Martin-Gallausiaux C. Marinelli L. Blottière H. M. Larraufie P. Lapaque N. (2021). Scfa: Mechanisms and functional importance in the gut. Proc. Nutr. Soc. 80 (1), 37–49. 10.1017/S0029665120006916 32238208

[B79] Mehta K. Laimins L. (2018). Human papillomaviruses preferentially recruit DNA repair factors to viral genomes for rapid repair and amplification. mBio 9 (1), 000644–e118. 10.1128/mBio.00064-18 PMC5821098 29440569

[B80] Miranda-Gonçalves V. Granja S. Martinho O. Honavar M. Pojo M. Costa B. M. (2016). Hypoxia-mediated upregulation of MCT1 expression supports the glycolytic phenotype of glioblastomas. Oncotarget 7 (29), 46335–46353. 10.18632/oncotarget.10114 27331625 PMC5216802

[B81] Miranda-Gonçalves V. Lameirinhas A. Macedo-Silva C. Lobo J. C Dias P. Ferreira V. (2020). Lactate increases renal cell carcinoma aggressiveness through sirtuin 1-dependent epithelial mesenchymal transition Axis regulation. Cells 9 (4), 1053. 10.3390/cells9041053 PMC7226526 32340156

[B82] Moitra K. (2015). Overcoming multidrug resistance in cancer stem cells. Biomed. Res. Int. 2015, 635745. 10.1155/2015/635745 26649310 PMC4663294

[B83] Monroe G. R. van Eerde A. M. Tessadori F. Duran K. J. Savelberg S. van Alfen J. C. (2019). Identification of human D lactate dehydrogenase deficiency. Nat. Commun. 10 (1), 1477. 10.1038/s41467-019-09458-6 30931947 PMC6443703

[B84] Morland C. Lauritzen K. H. Puchades M. Holm-Hansen S. Andersson K. Gjedde A. (2015). The lactate receptor, G-protein-coupled receptor 81/hydroxycarboxylic acid receptor 1: Expression and action in brain. J. Neurosci. Res. 93 (7), 1045–1055. 10.1002/jnr.23593 25881750

[B85] Moschen I. Bröer A. Galić S. Lang F. Bröer S. (2012). Significance of short chain fatty acid transport by members of the monocarboxylate transporter family (MCT). Neurochem. Res. 37 (11), 2562–2568. 10.1007/s11064-012-0857-3 22878645

[B86] Munakata Y. Saito-Ito T. Kumura-Ishii K. Huang J. Kodera T. Ishii T. (2005). Ku80 autoantigen as a cellular coreceptor for human parvovirus B19 infection. Blood 106 (10), 3449–3456. 10.1182/blood-2005-02-0536 16076874

[B87] Muylaert I. Elias P. (2007). Knockdown of DNA ligase IV/XRCC4 by RNA interference inhibits herpes simplex virus type I DNA replication. J. Biol. Chem. 282 (15), 10865–10872. 10.1074/jbc.M611834200 17296606

[B88] Naik A. Decock J. (2022). Targeting of lactate dehydrogenase C dysregulates the cell cycle and sensitizes breast cancer cells to DNA damage response targeted therapy. Mol. Oncol. 16 (4), 885–903. 10.1002/1878-0261.13024 34050611 PMC8847988

[B89] Nebenzahl-Sharon K. Shalata H. Sharf R. Amer J. Khoury-Haddad H. Sohn S. Y. (2019). Biphasic functional interaction between the adenovirus E4orf4 protein and DNA-PK. J. Virol. 93 (10), 013655–e1418. 10.1128/JVI.01365-18 PMC6498064 30842317

[B90] Nukuzuma S. Kameoka M. Sugiura S. Nakamichi K. Nukuzuma C. Takegami T. (2013). Suppressive effect of PARP-1 inhibitor on JC virus replication *in vitro* . J. Med. Virol. 85 (1), 132–137. 10.1002/jmv.23443 23074024

[B91] Ohno Y. Oyama A. Kaneko H. Egawa T. Yokoyama S. Sugiura T. (2018). Lactate increases myotube diameter via activation of MEK/ERK pathway in C2C12 cells. Acta Physiol. (Oxf) 223 (2), e13042. 10.1111/apha.13042 29377587

[B92] Papandreou I. Cairns R. A. Fontana L. Lim A. L. Dalko N. C. (2006). HIF-1 mediates adaptation to hypoxia by actively downregulating mitochondrial oxygen consumption. Cell Metab. 3 (3), 187–197. 10.1016/j.cmet.2006.01.012 16517406

[B93] Parada Venegas D. De la Fuente M. K. Landskron G. González M. J. Quera R. Dijkstra G. (2019). Short chain fatty acids (SCFAs)-Mediated gut epithelial and immune regulation and its relevance for inflammatory bowel diseases. Front. Immunol. 10, 277. 10.3389/fimmu.2019.00277 30915065 PMC6421268

[B94] Payen V. L. Mina E. Van Hée V. F. Porporato P. E. Sonveaux P. (2020). Monocarboxylate transporters in cancer. Mol. Metab. 33, 48–66. 10.1016/j.molmet.2019.07.006 31395464 PMC7056923

[B95] Perrine S. P. Hermine O. Small T. Suarez F. O'Reilly R. Boulad F. (2007). A phase 1/2 trial of arginine butyrate and ganciclovir in patients with Epstein-Barr virus-associated lymphoid malignancies. Blood 109 (6), 2571–2578. 10.1182/blood-2006-01-024703 17119113 PMC1852196

[B96] Peters N. E. Ferguson B. J. Mazzon M. Fahy A. S. Krysztofinska E. Arribas-Bosacoma R. (2013). A mechanism for the inhibition of DNA-PK-mediated DNA sensing by a virus. PLoS Pathog. 9 (10), e1003649. 10.1371/journal.ppat.1003649 24098118 PMC3789764

[B97] Petrova P. Petrov K. (2020). Lactic acid fermentation of cereals and pseudocereals: Ancient nutritional biotechnologies with modern applications. Nutrients 12 (4), 1118. 10.3390/nu12041118 PMC7230154 32316499

[B98] Phillips J. A. Griffin B. E. (2007). Pilot study of sodium phenylbutyrate as adjuvant in cyclophosphamide-resistant endemic Burkitt's lymphoma. Trans. R. Soc. Trop. Med. Hyg. 101 (12), 1265–1269. 10.1016/j.trstmh.2007.06.020 17915270

[B99] Pickard J. M. Zeng M. Y. Caruso R. Núñez G. (2017). Gut microbiota: Role in pathogen colonization, immune responses, and inflammatory disease. Immunol. Rev. 279 (1), 70–89. 10.1111/imr.12567 28856738 PMC5657496

[B100] Pinheiro C. Longatto-Filho A. Pereira S. M. Etlinger D. Moreira M. A. Jubé L. F. (2009). Monocarboxylate transporters 1 and 4 are associated with CD147 in cervical carcinoma. Dis. Markers 26 (3), 97–103. 10.3233/DMA-2009-0596 19597291 PMC3833680

[B101] Powell C. L. Davidson A. R. Brown A. M. (2020). Universal glia to neurone lactate transfer in the nervous system: Physiological functions and pathological consequences. Biosensors 10 (11), 183. 10.3390/bios10110183 PMC7699491 33228235

[B102] Qu J. Sun Z. Peng C. Li D. Yan W. Xu Z. (2021). *C. tropicali*s promotes chemotherapy resistance in colon cancer through increasing lactate production to regulate the mismatch repair system. Int. J. Biol. Sci. 17 (11), 2756–2769. 10.7150/ijbs.59262 34345205 PMC8326116

[B103] Raychaudhuri D. Bhattacharya R. Sinha B. P. Liu C. Ghosh A. R. Rahaman O. (2019). Lactate induces pro-tumor reprogramming in intratumoral plasmacytoid dendritic cells. Front. Immunol. 10, 1878. 10.3389/fimmu.2019.01878 31440253 PMC6692712

[B104] Reichardt N. Duncan S. H. Young P. Belenguer A. McWilliam Leitch C. Scott K. P. (2014). Phylogenetic distribution of three pathways for propionate production within the human gut microbiota. ISME J. 8 (6), 1323–1335. 10.1038/ismej.2014.14 24553467 PMC4030238

[B105] Roland C. L. Arumugam T. Deng D. Liu S. H. Philip B. Gomez S. (2014). Cell surface lactate receptor GPR81 is crucial for cancer cell survival. Cancer Res. 74 (18), 5301–5310. 10.1158/0008-5472.CAN-14-0319 24928781 PMC4167222

[B106] Shi B. Xu F. F. Xiang C. P. Jia R. Yan C. H. Ma S. Q. (2020). Effect of sodium butyrate on ABC transporters in lung cancer A549 and colorectal cancer HCT116 cells. Oncol. Lett. 20 (5), 148. 10.3892/ol.2020.12011 32934716 PMC7471751

[B107] Smith S. Reuven N. Mohni K. N. Schumacher A. J. Weller S. K. (2014). Structure of the herpes simplex virus 1 genome: Manipulation of nicks and gaps can abrogate infectivity and alter the cellular DNA damage response. J. Virol. 88 (17), 10146–10156. 10.1128/JVI.01723-14 24965466 PMC4136335

[B108] Soni V. K. Shukla D. Kumar A. Vishvakarma N. K. (2020). Curcumin circumvent lactate-induced chemoresistance in hepatic cancer cells through modulation of hydroxycarboxylic acid receptor-1. Int. J. Biochem. Cell Biol. 123, 105752. 10.1016/j.biocel.2020.105752 32325281

[B109] Taniguchi R. Utani K. Thakur B. Ishine K. Aladjem M. I. Shimizu N. (2021). SIRT1 stabilizes extrachromosomal gene amplification and contributes to repeat-induced gene silencing. J. Biol. Chem. 296, 100356. 10.1016/j.jbc.2021.100356 33539925 PMC7949162

[B110] Thomas S. P. Denu J. M. (2021). Short-chain fatty acids activate acetyltransferase p300. eLife 10, e72171. 10.7554/eLife.72171 34677127 PMC8585482

[B111] Trigg B. J. Lauer K. B. Fernandes Dos Santos P. Coleman H. Balmus G. Mansur D. S. (2017). The non-homologous end joining protein PAXX acts to restrict HSV-1 infection. Viruses 9 (11), 342. 10.3390/v9110342 PMC5707549 29144403

[B112] Uhl M. Csernok A. Aydin S. Kreienberg R. Wiesmüller L. Gatz S. A. (2010). Role of SIRT1 in homologous recombination. DNA Repair 9 (4), 383–393. 10.1016/j.dnarep.2009.12.020 20097625

[B113] Ullah M. S. Davies A. J. Halestrap A. P. (2006). The plasma membrane lactate transporter MCT4, but not MCT1, is up-regulated by hypoxia through a HIF-1alpha-dependent mechanism. J. Biol. Chem. 281 (14), 9030–9037. 10.1074/jbc.M511397200 16452478

[B114] Wagner W. Ciszewski W. Kania K. Dastych J. (2016). Lactate stimulates IL-4 and IL-13 production in activated HuT-78 T lymphocytes through a process that involves monocarboxylate transporters and protein hyperacetylation. J. Interferon Cytokine Res. 36 (5), 317–327. 10.1089/jir.2015.0086 27119568

[B115] Wagner W. Ciszewski W. M. Kania K. D. (2015). L- and D-lactate enhance DNA repair and modulate the resistance of cervical carcinoma cells to anticancer drugs via histone deacetylase inhibition and hydroxycarboxylic acid receptor 1 activation. Cell Commun. Signal. 13, 36. 10.1186/s12964-015-0114-x 26208712 PMC4514991

[B116] Wagner W. Kania K. D. Blauz A. Ciszewski W. M. (2017a). The lactate receptor (HCAR1/GPR81) contributes to doxorubicin chemoresistance via ABCB1 transporter up-regulation in human cervical cancer HeLa cells. J. Physiol. Pharmacol. 68 (4), 555–564. 29151072

[B117] Wagner W. Kania K. D. Ciszewski W. M. (2017b). Stimulation of lactate receptor (HCAR1) affects cellular DNA repair capacity. DNA Repair 52, 49–58. 10.1016/j.dnarep.2017.02.007 28258841

[B118] Wagner W. Sobierajska K. Kania K. D. Paradowska E. Ciszewski W. M. (2021). Lactate suppresses retroviral transduction in cervical epithelial cells through DNA-PKcs modulation. Int. J. Mol. Sci. 22 (24), 13194. 10.3390/ijms222413194 34947988 PMC8708659

[B119] Wang J. Kang L. Song D. Liu L. Yang S. Ma L. (2017). Ku70 senses HTLV-1 DNA and modulates HTLV-1 replication. J. Immunol. 199 (7), 2475–2482. 10.4049/jimmunol.1700111 28821586

[B120] Wang Y. Li H. Tang Q. Maul G. G. Yuan Y. (2008). Kaposi's sarcoma-associated herpesvirus ori-Lyt-dependent DNA replication: Involvement of host cellular factors. J. Virol. 82 (6), 2867–2882. 10.1128/JVI.01319-07 18199640 PMC2259006

[B121] Wang Z. H. Peng W. B. Zhang P. Yang X. P. Zhou Q. (2021). Lactate in the tumour microenvironment: From immune modulation to therapy. EBioMedicine 73, 103627. 10.1016/j.ebiom.2021.103627 34656878 PMC8524104

[B122] Westphal E. M. Blackstock W. Feng W. Israel B. Kenney S. C. (2000). Activation of lytic epstein-barr virus (EBV) infection by radiation and sodium butyrate *in vitro* and *in vivo*: A potential method for treating EBV-positive malignancies. Cancer Res. 60 (20), 5781–5788. 11059774

[B123] Xie G. Liu Y. Yao Q. Zheng R. Zhang L. Lin J. (2017). Hypoxia-induced angiotensin II by the lactate-chymase-dependent mechanism mediates radioresistance of hypoxic tumor cells. Sci. Rep. 7, 42396. 10.1038/srep42396 28205588 PMC5311966

[B124] Xu A. M. Huang P. H. (2010). Receptor tyrosine kinase coactivation networks in cancer. Cancer Res. 70 (10), 3857–3860. 10.1158/0008-5472.CAN-10-0163 20406984 PMC2875162

[B125] Yabu M. Shime H. Hara H. Saito T. Matsumoto M. Seya T. (2011). IL-23-dependent and -independent enhancement pathways of IL-17A production by lactic acid. Int. Immunol. 23 (1), 29–41. 10.1093/intimm/dxq455 21131367

[B126] Yang K. Chen Y. To K. K. Wang F. Li D. Chen L. (2017). Alectinib (CH5424802) antagonizes ABCB1- and ABCG2-mediated multidrug resistance *in vitro*, *in vivo* and *ex vivo* . Exp. Mol. Med. 49 (3), e303. 10.1038/emm.2016.168 28303028 PMC5382559

[B127] Yang K. Fan M. Wang X. Xu J. Wang Y. Tu F. (2022). Lactate promotes macrophage HMGB1 lactylation, acetylation, and exosomal release in polymicrobial sepsis. Cell Death Differ. 29 (1), 133–146. 10.1038/s41418-021-00841-9 34363018 PMC8738735

[B128] You D. Richardson J. R. Aleksunes L. M. (2020). Epigenetic regulation of multidrug resistance protein 1 and breast cancer resistance protein transporters by histone deacetylase inhibition. Drug Metab. Dispos. 48 (6), 459–480. 10.1124/dmd.119.089953 32193359 PMC7250367

[B129] Yu H. B. Jiang H. Cheng S. T. Hu Z. W. Ren J. H. Chen J. (2018). AGK2, A SIRT2 inhibitor, inhibits hepatitis B virus replication *in vitro* and *in vivo* . Int. J. Med. Sci. 15 (12), 1356–1364. 10.7150/ijms.26125 30275764 PMC6158674

[B130] Zeng L. Morinibu A. Kobayashi M. Zhu Y. Wang X. Goto Y. (2015). Aberrant IDH3α expression promotes malignant tumor growth by inducing HIF-1-mediated metabolic reprogramming and angiogenesis. Oncogene 34 (36), 4758–4766. 10.1038/onc.2014.411 25531325

